# Cloning of the cytochrome p450 reductase *(crtR) *gene and its involvement in the astaxanthin biosynthesis of *Xanthophyllomyces dendrorhous*

**DOI:** 10.1186/1471-2180-8-169

**Published:** 2008-10-06

**Authors:** Jennifer Alcaíno, Salvador Barahona, Marisela Carmona, Carla Lozano, Andrés Marcoleta, Mauricio Niklitschek, Dionisia Sepúlveda, Marcelo Baeza, Víctor Cifuentes

**Affiliations:** 1 Departamento de Ciencias Ecológicas, Facultad de Ciencias, Universidad de Chile, Santiago, Chile

## Abstract

**Background:**

The yeast *Xanthophyllomyces dendrorhous *synthesizes astaxanthin, a carotenoid with high commercial interest. The proposed biosynthetic route in this organism is isopentenyl-pyrophosphate (IPP) → geranyleranyl pyrophosphate (GGPP) → phytoene → lycopene → β-carotene → astaxanthin. Recently, it has been published that the conversion of β-carotene into astaxanthin requires only one enzyme, astaxanthin synthase or CrtS, encoded by *crtS *gene. This enzyme belongs to the cytochrome P450 protein family.

**Results:**

In this work, a *crtR *gene was isolated from *X. dendrorhous *yeast, which encodes a cytochrome P450 reductase (CPR) that provides CrtS with the necessary electrons for substrate oxygenation. We determined the structural organization of the *crtR *gene and its location in the yeast electrophoretic karyotype. Two transformants, CBSTr and T13, were obtained by deleting the *crtR *gene and inserting a hygromycin B resistance cassette. The carotenoid composition of the transformants was altered in relation to the wild type strain. CBSTr forms yellow colonies because it is unable to produce astaxanthin, hence accumulating β-carotene. T13 forms pale colonies because its astaxanthin content is reduced and its β-carotene content is increased.

**Conclusion:**

In addition to the *crtS *gene, *X. dendrorhous *requires a novel gene, *crtR*, for the conversion of β-carotene to astaxanthin.

## Background

Carotenoids are natural pigments of yellow, orange or red color. More than 600 different chemical structures have been described to date [1]. They are terpenoids with the isopentenyl- pyrophosphate (IPP) molecule as the basic unit. Astaxanthin is a carotenoid with a high commercial interest due to its use as a food additive for trout and salmon flesh pigmentation in aquaculture [2]. Its biosynthesis is limited to a few microorganisms such as the microalgae *Haematococcus pluvialis *and the basidiomycete yeast *Xanthophyllomyces dendrorhous *[3,4].

The first step in the biosynthesis of astaxanthin in *X. dendrorhous *is the isomerization of IPP into dimethylallyl pyrophosphate (DMAPP) by IPP isomerase, which is encoded by the *idi *gene [5]. Three IPP molecules are sequentially added to one DMAPP molecule, where the GGPP synthase (*crtE *gene) is involved, forming geranylgeranyl pyrophosphate (GGPP) [6]. Then, the condensation of two GGPP molecules produces phytoene, which corresponds to the first carotenoid synthesized in the pathway. This step is catalyzed by a bi-functional enzyme called phytoene-β-carotene synthase (PBS), which also has lycopene cyclase activity and is encoded by the *crtYB *gene [7]. Subsequently, phytoene goes through four successive desaturation reactions, catalyzed by the phytoene desaturase enzyme (*crtI *gene), producing lycopene [8]. Then, both lycopene ends are cyclized by the lycopene cyclase activity of the bi-functional PBS enzyme, forming β-carotene.

In the last step for the formation of astaxanthin from β-carotene, two enzymatic activities are required, ketolase, to incorporate a keto group in positions 4 and 4' of β-carotene, and hydroxylase, to introduce a hydroxyl group at positions 3 and 3' of β-carotene [9,10]. Unlike other organisms, where two independent genes are needed for this step, in *X. dendrorhous *a single *crtS *gene has been isolated that encodes an astaxanthin synthase which can probably perform both activities [11-13]. However, it has been suggested that CrtS may require an auxiliary enzyme for the formation of astaxanthin from β-carotene [14]. The deduced CrtS amino acid sequence strongly suggests that the *X. dendrorhous *enzyme belongs to the cytochrome P450 protein family [12,14].

Cytochrome P450 enzymes require an electron donor for the addition of oxygen-bearing functional groups to a substrate. In eukaryotes, this electron donor is cytochrome P450 reductase (CPR), encoded by the *cpr *gene [15]. Although several genes for different cytochrome P450 enzymes can exist in an organism, in most species only one *cpr *gene exists. Some exceptions have been observed in plants and zygomycetes that contain two or three *cpr *genes [16].

In another study, an *Escherichia coli *strain was transformed with three compatible plasmids. The first plasmid contained the necessary genes for the synthesis of β-carotene, the second contained the *X. dendrorhous crtS *gene, and the third contained the *Saccharomyces cerevisiae cpr *gene [12]. Through this system, it was possible to obtain oxygenated β-carotene derivatives, but not astaxanthin, suggesting the need for an appropriate CPR to complement the astaxanthin biosynthetic pathway. Prior to this study, a *X. dendrorhous *gene encoding for a CPR for astaxanthin biosynthesis had not been isolated. This paper describes for the first time the sequence of the *X. dendrorhous crtR *gene and its encoded polypeptide. Moreover, our results reveal that the *crtR *gene product is essential for astaxanthin biosynthesis in *X. dendrorhous*.

## Methods

### Strains, plasmids, media and enzymes

The strains and plasmids used in this study are listed in Tables 1 and 2. The wild type *X. dendrorhous *UCD 67–385 strain was used for genomic and cDNA library construction. *E. coli *DH-5α strain was used as a host for plasmid propagation and genomic libraries. Two genomic libraries were used in this study. The first library consisted of genomic DNA partially digested with *Bam*HI and cloned into the *Bam*HI site of the YIp5 vector [6]. The second library consisted of approximately 5 to 6 kb genomic DNA fragments digested with *Sal*I and cloned into the *Sal*I site of the pBluescript SK- vector. The cDNA library was constructed with the pBluescript II XR cDNA library construction kit from Stratagene [6].

**Table 1:** *E. coli *and *X. dendrorhous *strains used in this work

Strains	Genotype or relevant features	Reference
*E*. *coli*		
DH-5α	F^- ^φ80d *lac*ZΔM15Δ(*lac*ZY-*arg*F)U169 *deo*R *rec*A1 *end*A1 *hsd*R17(*r*_k_^- ^*m*_k_^+^) *pho*A *sup*E44l- *thi*-1 *gyr*A96 *rel*A1	[18]
DS1B	β-carotene producer strain with a yellow phenotype which corresponds to BL21-Gold strain carrying pDS1B	[6]
		
*X. dendrorhous*		
UCD 67–385	ATCC 24230, wild-type	ATCC
CBS-6938	ATCC 96594, wilt-type	ATCC
T13	(*crtR*^Δ*Bsi*WI::*hph*^/*crtR*^+^). Heterozygote transformant from UCD 67–385 parental wild-type strain, containing an allele of the *crtR *gene with a *Bsi*WI deletion and a hygromycin B resistance cassette.	This work
CBSTr	(*crtR*^Δ*Nde*I::*hph*^). Transformant from CBS-6938 parental wild-type strain. β-carotene accumulating mutant.	This work

**Table 2:** Plasmids used in this work

Plasmid	Genotype or relevant features	Source of reference
pBluescript SK-	ColE1 ori; Amp^R^; cloning vector with blue-white selection	Stratagene
pBAD33	p15 ori; Cam^R^; cloning vector	Beckwith Lab.
YIp5	*S. cerevisiae/E. coli *cloning vector. pMB1 ori; Amp^R^; TET^R^; URA3.	ATCC 37061
pET101/D-TOPO	ColE1 ori; Amp^R^; expression vector	Invitrogene
pDS1B	pBAD33 vector carrying the carotenogenesis genes *crtE*, *crtB*, *crtI *and *crtY *and *crtX *from *Erwinia uredovora *[40].	This work
pPR52.P.44	YIp5 bearing an insert of 12.8 kb that contains the *crtR *gene from base n° 88 of exon 1.	This work
pCPR1.3	pBluescript SK- bearing the DNA fragment from site *Bam*HI 4,529 to *Bam*HI 9,895 (Figure 1). pCPR1.3 was the *crtR *deletion platform.	This work
pcXDA.44.29	pBluescript SK- bearing the *crtR *gene cDNA. The cDNA measures 2,680 bp.	This work
pPR32.AA.51	YIp5 bearing an insert of 4.5 kb that contains the *crtR *gene up to base n° 87 of exon 1.	This work
pCPRB6	pBluescript SK- bearing an insert of 5.9 kb that contains the complete genomic version of *crtR *gene. Isolated from a *Sal*I partial genomic library.	This work
p*Bsi*WIΔ*crtR*::*hph*	pCPR1.3 *Bsi*WI deletion and *hph *cassette insertion.	This work
p*Nde*IΔ*crtR*::*hph*	pCPR1.3 *Nde*I deletion and *hph *cassette insertion.	This work

*crtR *gene deletion bearing plasmids (p*Bsi*WIΔ*crtR*::*hph *and p*Nde*IΔ*crtR*::*hph*) were built from pCPR1.3 (Table 2), which has the *X. dendrorhous crtR *gene. The deletions were created by digesting pCPR1.3 with *Bsi*WI or *Nde*I and then filling the ends with Klenow polymerase to enable the ligation of the blunt ended hygromycin B resistance cassette (*hph*) [6] for the selection of *X. dendrorhous *transformants.

*X. dendrorhous *strains were grown at 22°C with constant agitation in YM medium (1% glucose, 0.3% yeast extract, 0.3% malt extract and 0.5% peptone) or MM_V _+ 2% glucose medium [17]. UCD 67–385 strain fermentation was performed in a 12 l jar fermentor (New Brunswick) containing 8.8 l of MM_V _+ 2% glucose medium, shaking at 300 rpm and 13 l/min sterile air injection. Antifoam agent (1520 US, Dow Corning) was automatically added when required. *E. coli *strains were grown with constant agitation at 37°C in Luria-Bertani (LB) medium and supplemented with 100 μg/ml ampicillin for plasmid selection and 40 μl of a 2% solution of X-gal (5-bromo-4chloro-3-indolyl-β-D-galactopyranoside) for recombinant clone selection [18]. For *E. coli *complementation experiments, the LB growth medium was supplemented with 10 μM hemine and 0.5 mM δ-aminolevulinic acid, which supports heme prosthetic group formation in *E. coli *[12,19]. The expression was induced with 1 mM IPTG. Enzymes were purchased from Promega (*Taq*DNA pol, restriction enzymes, Klenow polimerase, M-MLV reverse transcriptase).

### DNA amplification

Primers were purchased from Alpha DNA (Montreal, Canada) and are listed in Table 3. PCR reactions were performed in a final volume of 25 μl containing 2 U of *Taq*DNA pol, 2.5 μl of 10× Taq buffer, 0.5 μl of 10 mM dNTPs, 1 μl of 50 mM MgCl_2_, 1 μl of 25 μM of each primer and 10–20 ng DNA. Amplification was done in a DNA thermal cycler 2400 (Perkin-Elmer) as follows: initial denaturation at 95°C for 3 min; 35 cycles of denaturation at 94°C for 30 s, annealing at 55°C for 30 s, synthesis at 72°C for 3 min and a final extension step at 72°C for 10 min. Samples were kept at 4°C until checked. The amplicons were separated by 0.8% agarose gel electrophoresis in TAE buffer containing 0.5 μg/ml ethidium bromide [18] followed by DNA purification using glassmilk [20] for sequencing.

**Table 3:** Primers used in this work

Primer	Direction	Sequence 5' to 3'	Target
CPR3	F	CARACTGGKACDGCHGARGATT	*crtR *gene
CPR4	F	CAAACTGGTACGGCTGAAGATT	*crtR *gene
CPR5	R	WGGDCCRATCATGAYRACTGG	*crtR *gene
CPR6	R	AGGTCCAATCATGACGACTGG	*crtR *gene
CPR7	R	CRGTACCWGGDCCRATCATGA	*crtR *gene
CPR8	R	CCAGTACCAGGTCCAATCATGA	*crtR *gene
CPR9	F	GGATCCGCGACATCGAAGAGTATGAC	*crtR *gene
CPR12	R	GGATCCCTTCCAAGCGAGGTAGTCTT	*crtR *gene
CPR13	R	AGAAGACTGTGCGATCGTGT	*crtR *gene
CPR31	R	TCAAGCAATTGGTGTTGGTC	*crtR *gene
CPR56	R	TCGTTGTGTTGTTGCTATTCG	*crtR *gene
CPR3A	F	TACAACGTCGTCGGTAGACA	*crtR *gene
CPR7A	R	TATTCCACACCGTGGTGTTC	*crtR *gene
CPR3D	F	GGTCTCACTTCTCCAGAGAA	*crtR *gene
CPRfwdC	F	GCACAGGAAGTTGGTTGGAT	*crtR *gene
CPR1exC	F	GCCACACTCTCCGATCTTGT	*crtR *gene
CPREx1fwdb	F	TCAGGACCCTGTACAGTCAGC	*crtR *gene
CPREx1rev	R	TCCCAACAGTCGATCCTTGT	*crtR *gene
Pef	F	GATATCGGCTCATCAGCCGAC	EF-1α Promoter
Gpd	R	ATGAGAGATGACGGAGATG	GPDH Terminator
HR	R	CTATTCCTTTGCCCTCGGAC	*hph *gene
HF	F	ATGAAAAAGCCTGAACTCACC	*hph *gene
HygSecR	R	GTATTGACCGATTCCTTGCG	*hph *gene
HygSecF	F	TCGCCAACATCTTCTTCTGG	*hph *gene
M13R	R	GGAAACAGCTATGACCATG	pBluescript SK-
M13F	F	TGTAAAACGACGGCCAGT	pBluescript SK-
ACT3	F	ACTCCTACGTTGGTGACGAG	Actin gene
ACT4	R	TCAAGTCTCGACCGGCCAAG	Actin gene
AST1	F	GCCACCTACTTTCTCCATATGT	*crtS *gene
AST2	R	GAGCCATGACGTCCAGAGTA	*crtS *gene

F: forward, R: reverse

### DNA sequence and bioinformatic tools

Nucleotide sequences were obtained from an ABI 3100 Avant genetic analyzer, using a DYEnamic ET terminator Kit (Amersham Bioscience). DNA sequences were analyzed with Vector NTI Suite 10 (Informax), CLUSTAL W 1.8 and BLAST programs. Protein analysis was performed with the online programs InterProScan  and TMPRED . Phylogenetic analysis was carried out using BioEdit 7.0.0 and Treecon 1.3b.

### Pulse field gel electrophoresis

Chromosomal DNA was separated by contour-clamped homogeneous electric fields (CHEF) in a CHEF II BioRad system in 0.9% agarose gels in TBE 0.5× buffer (45 mM Tris-borate, 1 mM EDTA pH 8.0) at 14°C [21]. The pulses used were 90 s for 24 h, followed by 120 s for 24 h at 6 V/cm.

### Southern blot hybridization

Southern blot hybridization was performed according to Sambrook and Russell [18]. *crtR *probe was obtained by PCR amplification with primers CPR3 and CPR7 of the *crtR *gene. *hph *probe was acquired from PCR amplification of the *hph *cassette using primers HF and HR. Probes were labeled with [^32^P]α-dCTP using the Promega prime-a-gene labeling system.

### RNA extraction and cDNA synthesis

Total RNA extraction was performed according to a modified protocol of Chomczynski and Sacchi [22,23]. The determination of the relative levels of *crtS *and *crtR *mRNAs was performed using a semi-quantitative RT-PCR method. The intensities of the *crtS *and *crtR *amplification bands were normalized with the intensity of the actin [24] amplification product [23,25]. The amplicons were quantified with ImageJ 1.40 using a 100 bp DNA ladder (Fermentas) as the standard.

### *X. dendrorhous *transformation

*X. dendrorhous *transformation was performed by electroporation according to [26] and [27]. Electrocompetent cells were prepared from an exponential culture with DO_600 nm _= 1.2 grown in YM medium [6] and electroporated using a BioRad gene pulser × cell with PC and CE modules under the following conditions: 125 mF, 600 Ω, 0.45 kV. Transformant selection was performed in YM 1.5% agar plates supplemented with 10 μg/ml hygromycin B. The transformants were identified as *X. dendrorhous *by ITS1, 5.8 rRNA gene and ITS2 DNA sequences [28].

### Pigment extraction and RP-HPLC

Carotenoid extraction was carried out from cellular pellets according to the acetone extraction method [29]. Carotenoids were quantified by absorbance at 465 nm using an absorption coefficient of A_1% _= 2,100. The analyses were performed in triplicate, and pigments were normalized relative to the dry weight of the yeast. Carotenoids were separated by RP-HPLC using a reverse phase RP-18 Lichrocart 125-4 (Merck) column with acetonitrile: methanol: isopropyl (85:10:5 v/v) as the mobile phase with a 1 ml/min flux under isocratic conditions. The elusion spectra were recuperated using a diode array detector.

## Results and discussion

### Cloning of *crtR* gene

Three cytochrome P450 reductase genes were used for primer design: from the zygomycete *Cunninghamella echinulata *[GenBank:AF195660], the ascomycete *Saccharomyces cerevisiae *[GenBank:D13788] and the basidiomycete *Rhodotorula minuta *[GenBank:AB055119]. Based on the conserved DNA segments, degenerate primers were designed (Table 3). PCR reactions were performed using genomic DNA from UCD 67–385 *X. dendrorhous *as a template and different combinations of forward and reverse primers. An amplicon of 1.6 kb was obtained when PCR reactions were performed with primers CPR3 and CPR7. This amplicon was completely sequenced and was found to be homologous to CPR genes by BLAST analysis. We named this gene *crtR *and designed specific *crtR *primers.

The YIp5 genomic library was screened for the *X. dendrorhous crtR *gene by PCR [6], and one recombinant plasmid carrying the *crtR *gene (pPR52.P.44) was isolated. This plasmid contained a DNA insert of approximately 12.8 kb, carrying six *Bam*HI fragments of 5.4 kb, 4.4 kb, 0.9 kb (doublet), 0.8 kb and 0.4 kb. Each fragment was sub-cloned into the pBluescript SK- vector for sequencing. The sequence analysis showed that the *crtR *gene was in one end of the 5.4 kb fragment (pCPR1.3), but it was incomplete. The promoter region and part of the first exon were missing due to the presence of a *Bam*HI site in the *crtR *gene.

A cDNA library was screened for the *X. dendrorhous crtR *cDNA, and a recombinant plasmid, pcXDA.44.29, was isolated, which contained a 2,680 bp insert corresponding to the cDNA of the *crtR *gene [GenBank:EU884134]. We used this cDNA sequence for specific primer design (primers CPREx1fwdb and CPREx1rev, Table 3) from the remaining *crtR *gene sequence, and this allowed the isolation of the pPR32.AA.51 plasmid from the YIp5 genomic library. pPR32.AA.51 contains the promoter and 5' region of the first exon of the *crtR *gene. Sequence analysis indicated that the 5' region of the *crtR *gene is at one end of the 4.5 kb *Bam*HI DNA fragment.

**Figure 1:**
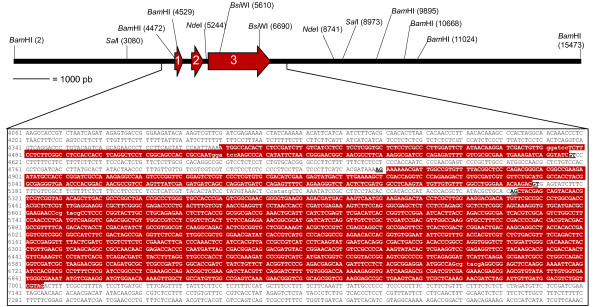
**Graphical representation and sequence of the *crtR* gene from *X. dendrorhous***. The three *crtR *exons are highlighted in red and white letters. The restriction enzymes used in this work and their recognition sites are in parentheses. Underlined and bold black letters represent the donor and acceptor splice sites. The sequence is numbered in accordance with its graphical representation. The complete sequence of the DNA fragment that contains the *crtR *gene is in GenBank [GenBank:EU884133]. pCPR1.3 corresponds to the vector pBluescript SK- bearing the DNA fragment from site *Bam*HI 4529 to *Bam*HI 9895. *crtR *deletions were constructed from pCPR1.3.

The *in silico *DNA sequence analysis of the inserts from pPR32.AA.51 and pPR52.P.44 indicated that the *crtR *gene is entirely contained in a 5.9 kb *Sal*I DNA fragment. This result was corroborated by Southern blot hybridization (data not shown). The recombinant plasmid pCPRB.6, which contains an insert of 5,896 bp that harbors the *crtR *gene, was isolated from a partial *Sal*I genomic library. The complete *crtR *gene sequence [GenBank:EU884133] is displayed in Figure 1.

### Localization of the *crtR* gene in the yeast electrophoretic karyotype

**Figure 2:**
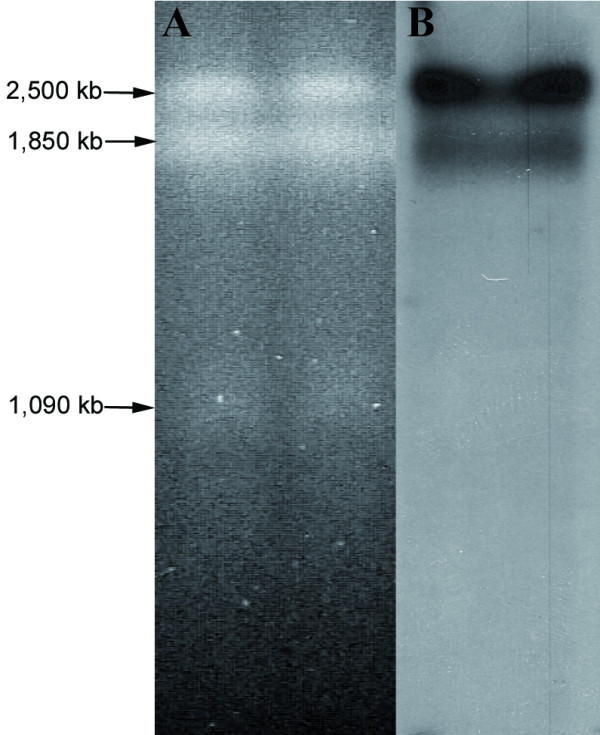
***X. dendrorhous *electrophoretic karyotype**. (A) CHEF of *X. dendrorhous *UCD 67–385 strain. (B) Hybridization with *crtR *probe.

To study the *crtR *gene's genomic organization, its localization in the electrophoretic karyotype of the UCD 67–385 strain was performed by Southern blot hybridization. The results showed that the *crtR *gene is in the first two chromosomal bands (Figure 2), where each band corresponds to a triplet [21]. As it was recently demonstrated that this strain is diploid [30], it is possible to conclude that the *crtR *gene is in at least two chromosomes and could be homologous polymorphic. In different *X. dendrorhous *strains, a high chromosomal polymorphism has been revealed [31], so it is possible that these homologous chromosomes have experienced genetic rearrangement, thus explaining their different sizes.

### Sequence analysis of *crtR *gene

**Figure 3:**
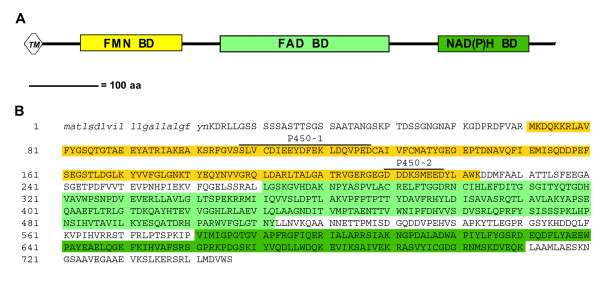
**Deduced amino acid sequence from *X. dendrorhous *CrtR**. (A) Graphical representation of CPR conserved domains in *X. dendrorhous *CrtR. (B) Deduced CrtR protein sequence. Conserved domains are shown in white text. TM: in italics and lower case. Highlighted in yellow: FMN BD, green: FAD BD and dark green: NAD(P)H BD. The two acidic regions involved in the binding to cytochrome P450 are also shown. *TM*: transmembrane region, BD: binding domain.

The *crtR *gene consists of three exons of 222, 317 and 1702 bp and two introns of 240 and 138 bp. This gene encodes a 746 amino acid protein (Figure 3) with a predicted molecular weight of 81.9 kDa. CrtR has 22 hydrophobic amino acids in its amino terminal region, a typical transmembrane segment of known CPRs that anchors the protein to the endoplasmic reticulum membrane in the cell [32]. CrtR also has recognizable CPR conserved domains such as a FMN binding domain, a FAD binding domain between amino acids 275 and 512, and a NAD(P)H binding domain between amino acids 582 and 709. The flavodoxin signature, which is only found in certain bacteria and algae, was identified between amino acids 80 and 222. However, the FMN binding domain of CPRs is homologous to bacterial flavodoxins [33]. Two putative P450-binding regions (P450-1 and P450-2) have been suggested [32], and one (P450-2) was studied by site-directed mutagenesis in rat CPR where the importance of acidic amino acid residues in this region was demonstrated [34]. The corresponding regions in CrtR were characterized by the presence of acidic amino acids (aspartic acid at positions: 112, 117, 122, 127, 210, 211, 218; glutamic acid at positions: 114, 115, 119, 126, 216, 217). The aspartic acid residues at position 210, 211 and 218 are extremely conserved in all known CPRs [32]. The P450 binding regions are located in the FMN binding domain. This is consistent with the electron flow from NAD(P)H to FAD to FMN and finally to the cytochrome P450 monooxygenase enzyme.

**Figure 4:**
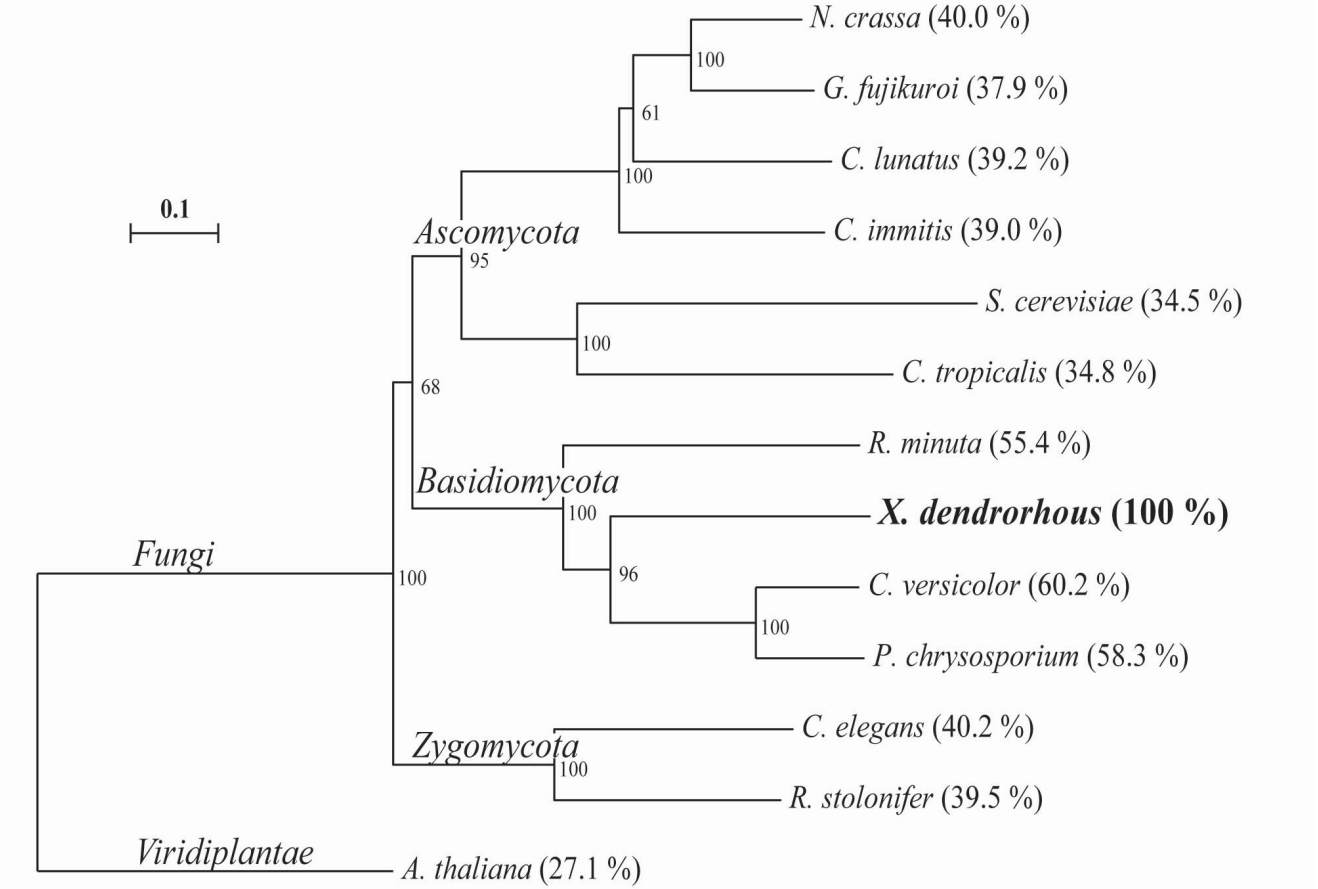
**Phylogenetic tree for cytochrome P450 reductases**. Distance analysis and tree construction was done using the Neighbor Joining method. Numbers at nodes indicate the percent support for specific nodes after 1000 replications of bootstrap analysis. The scale bar indicates 10% change estimated sequence difference. The percentage of sequence identity with *X. dendrorhous *CrtR is in parenthesis. Accession numbers: *Neurospora crassa*: [GenBank:XP_964443]; *Gibberella fujikuroi*: [GenBank:AJ576025]; *Cochliobolus lunatus*: [GenBank:ABW86977]; *Coccidioides immitis*: [GenBank:XP_001246449]; *Saccharomyces cerevisiae*: [GenBank:D13788]; *Candida tropicalis*: [GenBank:M35199]; *Rhodotorula minuta*: [GenBank:AB055119]; *Xanthophyllomyces dendrorhous*: [GenBank:EU884134] and [GenBank:EU884133]; *Coriolus versicolor*: [GenBank:AB065368]; *Phanerochaete chrysosporium*: [GenBank:AF193060]; *Cunninghamella elegans*: [GenBank:AF195659]; *Rhizopus stolonifer*: [GenBank:AF290425]; *Arabidopsis thaliana*: [GenBank:A75959].

A phylogenetic comparison was performed using available fungal CPR protein sequences in the database by the Neighbor Joining method after 1000 replications of bootstrap analysis (Figure 4). The phylogenetic tree shows the evolutionary relationship of CrtR and allows the differentiation of three fungal clusters: Basidomycota, Ascomycota and Zygomycota. CrtR from *X. dendrorhous *is most closely related to the basidomycetes *C. versicolor *and to *P. chrysosporium*, with sequence identities of 60.2% and 58.3%, respectively.

### Expression of *crtR *gene

**Figure 5:**
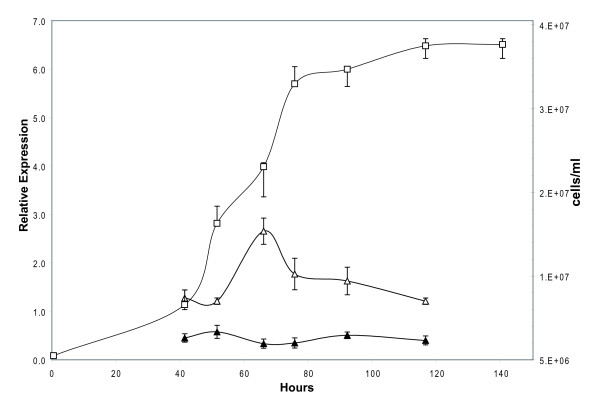
**Relative expression of *crtR *and *crtS *genes along the *X. dendrorhous *growth curve**. Wild type UCD 67–385 strain was grown at 22°C in a fermentor with MM_V _+ 2% glucose medium. (Δ): *crtS *relative expression, (△): *crtR *relative expression and (▲): n° cells per ml.

The relationship between the *crtS *and *crtR *gene expression along the growth curve at the mRNA level was studied in the wild type UCD 67–385 *X. dendrorhous *strain, grown in MM_V _supplemented with glucose as a fermentable carbon source. It was observed that *crtS *mRNA level reaches a maximum at 66 h of cultivation, equivalent to approximately half of the exponential growth phase. However, the levels of *crtR *mRNA remained constant along the growth curve (Figure 5). Despite the fact that both proteins are involved in the same stage of astaxanthin biosynthesis, there is no relationship, at the mRNA level, in their gene expression. It is possible to conclude that the two genes are not regulated in the same way at the transcriptional level.

According to this last result, in other work done in our laboratory, a dramatic decrease of transcripts levels from the *crtYB*, *crtI *and *crtS *genes (carotenogenic genes) was observed during the stationary phase [35]. This was not the case for the *idi *and *crtE *genes, where the level of the *idi *transcript showed a slight decline and the *crtE *transcript remained constant during the stationary phase. This suggests the presence of higher levels of the enzymes IPP isomerase and GGPP synthase at this stage. This situation would allow for the flow of the isoprenoid precursors towards carotenoid biosynthesis, as well as other metabolic pathways such as ergosterol biosynthesis [35]. Similarly, the absence of a relationship between the pattern of *crtR *gene expression and the rest of the carotenogenic genes suggests the existence of a different regulatory mechanism for *crtR *gene expression due to its involvement in other metabolic pathways of the yeast.

An example of *cpr *gene regulation has been described for *Aspergillus niger*. In this case, it was observed that the regulation of *cpr *gene expression was particularly complex in the P450-CPR system involved in benzoate hydroxylation [36]. In addition to benzoate induction at the transcriptional level, there were other regulatory mechanisms such as differential promoter use and post-translational regulatory mechanisms [36]. The decoupling of a P450-CPR system would imply the release of electrons to the cytoplasm that could generate active oxygen molecules, so a strict regulation of CPR is of vital importance for the cell. Similarly, it is not surprising that the *crtS *and *crtR *genes are regulated differently because, as mentioned earlier, several genes for different cytochrome P450 enzymes can exist in an organism, but generally there is a single gene for a unique cytochrome P450 reductase. Therefore, complex regulation for CrtR protein expression is necessary to adjust the levels of cytochrome P450 reductase activity in a cell with different levels of diverse cytochrome P450 proteins.

### Functional study of the *crtR *gene

The participation of the *crtR* gene product in the formation of astaxanthin from β-carotene was studied in a bacterial heterologous system. For this, the DS1B *E. coli* strain (Table 1) was transformed with a pET101/D-TOPO expression vector carrying the cDNA of *crtS* and *crtR* genes from *X. dendrorhous*, and the carotenoid production was analyzed by RP-HPLC. This system was not successful, as it was not possible to produce any xanthophylls in the bacteria. A similar situation was observed in another study, where only canthaxanthin (β-carotene-4, 4'-dione, which is an intermediate in the conversion of β-carotene into astaxanthin) in very small quantities was produced when the *S. cerevisiae cpr *gene was used [12]. These authors concluded that the *X. dendrorhous *astaxanthin synthase has very low activity in *E. coli *[12]. It should be noted that the P450 systems are associated with membranes; therefore, *E. coli *may not provide a proper environment for its functionality. Recently, it was reported that several attempts to produce carotenoids in *E. coli *using the *X. dendrorhous *carotenogenic genes resulted in poor enzyme expression and carotenoid production [37]. Hence, a eukaryotic system would be more appropriate to attempt the expression of *X. dendrorhous *carotenogenic genes.

**Figure 6:**
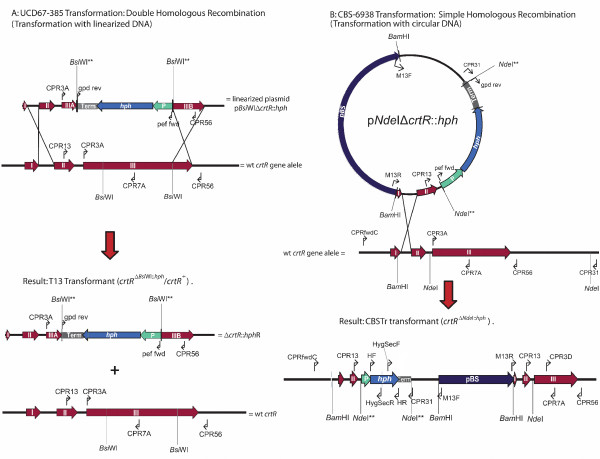
**Graphical representation of transformation events**. Wild type strains UCD 67–385 and CBS-6938 were transformed with the linearized plasmid p*Bsi*WIΔ*crtR*::*hph *and circular plasmid p*Nde*IΔ*crtR*::*hph*, respectively. I, II, III: represent *crtR *exons 1, 2 and 3; P: EF-1α promoter, Term: GPDH transcription terminator; *hph*: hygromycin B resistance gene; pBS: pBluescript; *Bsi*WI** and *Nde*I**: modified restriction sites after hygromycin B cassette insertion and arrows represent primer binding sites.

As the *E. coli *expression system was not successful, in order to demonstrate the importance of the *crtR *gene in the astaxanthin biosynthetic pathway, *X. dendrorhous crtR *deletion mutant strains were generated. The wild type strains UCD 67–385 and CBS-6938 were transformed with the linearized plasmid p*Bsi*WIΔ*crtR*::*hph *and circular plasmid p*Nde*IΔ*crtR*::*hph*, respectively. Through homologous recombination events, the wild type *crtR *gene in the wild type strains was exchanged by the DNA fragment containing the deletion and resistance marker (Figure 6).

Through this experiment, a yellow (CBSTr, which derives from CBS-6938) and a pale red-orange (T13, which derives from UCD 67–385) hygromycin B resistant transformant strains were obtained. The color phenotype of these transformants is an indicator of an alteration in astaxanthin biosynthesis because astaxanthin has a strong red-orange color. The strain UCD 67–385 is diploid [30]; therefore, we suggest that T13 is heterozygote for the *crtR *gene, with a mutant and a wild type allele. Moreover, by a gene-dose effect, T13 produces less astaxanthin and accumulates more β-carotene than its parental wild type strain. On the other hand, the ploidy level of CBS-6938 is unknown. However, based on random mutagenesis results with physical and chemical mutagens performed in our laboratory (data not shown) and transformation with carotenogenic genes [38], it was concluded that this strain could be haploid. The mutagenesis results suggest that, in the case of CBSTr, the only *crtR *gene copy is mutated and, therefore, it is not capable of producing astaxanthin and accumulates β-carotene.

In order to confirm the T13 (*crtR*^Δ*Bsi*WI::*hph*^/*crtR*^+^) and CBSTr (*crtR*^Δ*Nde*I::*hph*^) transformant genotype modifications, PCR reactions were performed using specific primers for the *crtR *and *hph *genes (Table 2), and genomic DNA samples from wild type and transformant strains were used as templates. The amplicon sequences validated the representation shown in Figure 6. The results indicated that the CBS-6938 strain has one *crtR *gene copy and its deletion is not lethal. In the case of T13, it was shown that it is heterozygote for the *crtR *gene, having a wild type and a mutant allele, which supports the gene-dose effect hypothesis in the production of astaxanthin. These results were also corroborated by Southern blot hybridization (data not shown).

Several attempts were performed to obtain a β-carotene accumulating transformant derived from UCD 67–385. T13 was retransformed with a DNA fragment harboring a *crtR *gene deletion and a G418 resistance cassette marker. No G418 resistant transformants were obtained. In order to produce a homozygous *crtR *mutation, T13 was subjected to the Double Recombinant Method [6], but it was not possible to obtain a double mutant strain for the *crtR *gene.

The *cpr *gene deletion in *S. cerevisiae *was not lethal, suggesting the existence of an alternative electron donor such as a cytochrome b5 (*cytb5*) [39]. When a *cytb5 *deletion in the wild type was done, no new phenotype was generated. However, it was lethal when *cytb5 *and *cpr *were simultaneously disrupted. This result demonstrated that, in mutants with single disruptions of *cpr *or *cytb5*, both enzymes can complement each other [39]. Likewise, the disruption of the *cpr *gene from the fungus *Gibberella fujikuroi *was not lethal, proving the existence of another electron donor [16]. In light of these results and evidence for the existence of a high level of polymorphisms in different *X. dendrorhous *strains [21,30,31], we suggest that both wild type strains may have a different genetic background. Moreover, the results obtained in this work suggest that, in the CBS-6938 wild type strain, an alternative electron donor such as a cytochrome b5 may exist because the *crtR *gene disruption was not lethal. On the other hand, this is not the case for the UCD 67–385 strain due to the impossibility to obtain a homozygous mutant for the *crtR *gene. However, the possible alternative electron donor in the CBS-6938 strain cannot support astaxanthin synthesis, as the *crtR *gene mutant (CBSTr) accumulates β-carotene.

**Figure 7:**
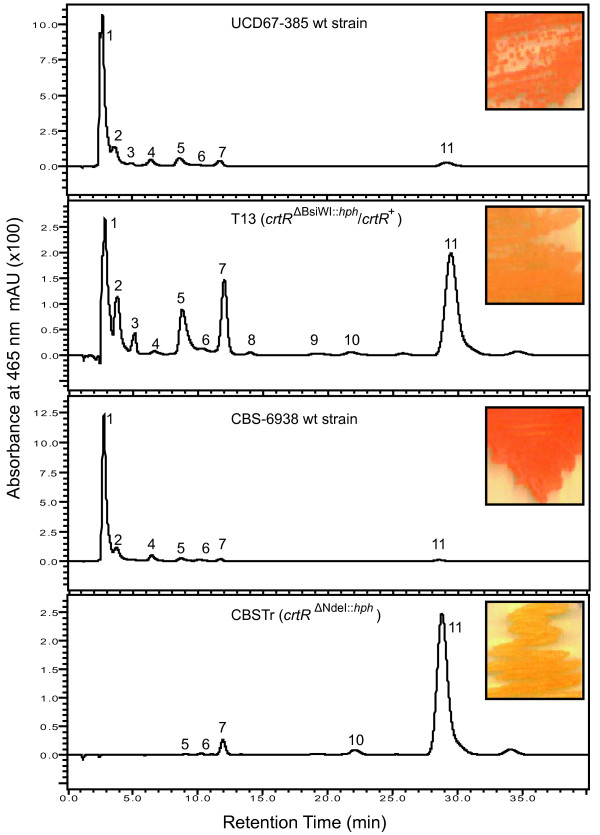
**RP-HPLC analysis of carotenoids from wild type (UCD 67–385, CBS-6983) and transformant (T13 (*crtR*^Δ*Bsi*WI::*hph*^/*crtR*^+^), CBSTr (*crtR*^Δ*Nde*I::*hph*^)) strains**. Numbers indicate different carotenoids as follows: 1: astaxanthin; 2: phoenicoxanthin; 3: canthaxanthin; 4: hydroxy-keto-γ-carotene; 5: keto-γ-carotene; 6: hydroxy-echinenone; 7: echinenone; 8: lycopene; 9: neurosporene; 10: γ-carotene and 11: β-carotene. In the upper right of the chromatogram, colony phenotype is shown.

To check the carotenoid composition, total pigments were extracted from both wild type and transformant strains, and the composition was analyzed by RP-HPLC (Figure 7 and Table 4). The RP-HPLC analysis showed that the carotenoid composition is completely different between transformants and their parental strain. CBSTr accumulates β-carotene and is unable to synthesize astaxanthin. T13 has a reduced astaxanthin production at the expense of increased β-carotene production, which is the astaxanthin precursor.

**Table 4:** Carotenoid composition of wild-type and transformant *X. dendrorhous *strains in ppm

**Carotenoid**	**UCD 67–385 wt-strain**	**T13 (*crtR*^Δ*Bsi*WI::*hph*^/*crtR*^+^)**	**CBS-6938 wt-strain**	**CBSTr (*crtR*^Δ*Nde*I::*hph*^)**
astaxanthin	102.0 ± (4.8)	24.0 ± (3.6)	152.0 ± (8.5)	ND
phoenicoxanthin	7.0 ± (0.4)	8.0 ± (0.4)	8.0 ± (0.5)	ND
canthaxanthin	1.0 ± (0.1)	3.00 ± (0.06)	1.0 ± (0.1)	ND
hydroxy-keto-γ-carotene	6.0 ± (0.2)	1.00 ± (0.06)	ND	ND
HO-keto-torulene	ND	ND	6.0 ± (1.1)	ND
keto-γ-carotene	9.0 ± (0.7)	11.0 ± (0.7)	8.0 ± (1.2)	1.0 ± (0.3)
HO-echinenone	ND	ND	2.0 ± (0.4)	1.0 ± (0.4)
echinenone	5.0 ± (0.6)	16.0 ± (0.5)	5.0 ± (0.7)	8.0 ± (1.2)
lycopene	ND	1.0 ± (0.01)	ND	ND
neurosporene	ND	1.00 ± (0.02)	ND	ND
γ-carotene	1.0 ± (0.1)	2.00 ± (0.05)	1.0 ± (0.1)	3.0 ± (0.2)
β-carotene	9.0 ± (1.7)	50.0 ± (3.7)	9.0 ± (1.5)	114.5 ± (6.6)
phytoene	0.4 ± (0.3)	4.2 ± (1.9)	3.3 ± (1.2)	1.0 ± (0.4)
NI (25 min)*	ND	1.0 ± (0.1)	ND	1.0 ± (0.1)
NI (34 min)*	ND	2.00 ± (0.04)	ND	4.0 ± (0.9)
Total carotenoids	139.0 ± (3.2)	122.8 ± (8.0)	195.3 ± (8.0)	131.9 ± (7.5)

ND: Not detected, NI: Not identified carotenoid. Table values are the average results of three independent experiments, SD in parenthesis. ()*: NI retention time.

## Conclusion

A new *crtR *gene has been isolated and characterized from *X. dendrorhous*, which plays an auxiliary role to the *crtS *gene and is also essential for astaxanthin biosynthesis. This gene consists of three exons, and is localized in at least two chromosomes of the UCD 67–385 *X. dendrorhous *wild type strain. *crtR *encodes a 746 amino acid protein with characteristic domains of cytochrome P450 reductases (FMN, FAD and NAD(P)H binding domains) and a small amino terminal transmembrane region. Although both *crtS *and *crtR *proteins participate in the same stage of yeast carotenogenesis, the mRNA level pattern from these genes along the growth curve is different. Thus, both genes are regulated in a different manner at the transcriptional level.

## Authors' contributions

JA participated in the design of this study and carried out the *crtR *gene isolation, sequence analysis, *crtR *gene expression study, *X. dendrorhous *transformation, pigment extraction, RP-HPLC and drafted the manuscript. SB constructed the *X. dendrorhous *YIp5 genomic library. MC participated in the isolation of the *crtS *gene genomic and cDNA versions. CL participated in the UCD 67–385 strain fermentation and RT-PCR analysis. AM constructed the *X. dendrorhous *cDNA library. MN constructed the hygromycin B resistance cassette. DS participated in DNA sequencing. MB participated in the study design. VC conceived the study and participated in its design and coordination. All authors read and approved the final manuscript.
